# Association between serum albumin and severe impairment of activities of daily living in patients with stroke: a cross-sectional study

**DOI:** 10.3389/fneur.2024.1501294

**Published:** 2025-01-06

**Authors:** Ranran Bi, Yupeng Shi, Manrong Li, Xiaochen Liu, Zhenchao Ma, Yiqing Huang, Bingyin Liang, Fang Cui

**Affiliations:** ^1^Department of Rehabilitation Medicine, Shanghai East Hospital, School of Medicine, Tongji University, Shanghai, China; ^2^Shandong Provincial Key Medical and Health Laboratory of Intensive Care Rehabilitation, Shandong Provincial Third Hospital, Cheeloo College of Medicine, Shandong University, Jinan, China

**Keywords:** serum albumin, severe impairment, activities of daily living, stroke, L-shaped, cross-sectional study

## Abstract

**Purpose:**

The relationship between serum albumin levels and severe limitations in ADLs among stroke patients remains unclear. Specifically, the dose–response relationship between the two needs further exploration. This study aims to provide further results.

**Materials and methods:**

This study examined cross-sectional data from patients aged 18 years or older with a diagnosis of stroke confirmed by cranial CT or MRI within 24 h of admission, gathered from January 2020 to August 2022. Data included serum albumin levels, Barthel Index scores recorded after admission, and other essential variables.

**Results:**

The study comprised 2,393 stroke patients. After adjusting for confounding factors, the multivariate analysis revealed a 7% decrease in severe impairment of ADL after stroke for every unit (g/L) increase in serum albumin levels. Compared with individuals with lower serum albumin levels (Q1: ≤ 37.4 g/L), the adjusted odds ratios (OR) for severe of ADL impairment among stroke patients in Q2 (37.4–40.21 g/L), Q3 (40.21–42.80 g/L), and Q4 (≥42.8 g/L) were 0.68 (95% CI: 0.4–1.15, *p* = 0.148), 0.55 (95% CI: 0.32–0.97, *p* = 0.04), and 0.64 (95% CI: 0.37–1.15, *p* = 0.139), respectively. The relationship between serum albumin and severe impairment of ADLs in stroke patients showed an L-shaped curve (non-linear, *p* = 0.002), with an inflection point at 38.0 g/L. The OR for significant impairment of ADLs was 0.680 (95% CI: 0.568–0.814, *p* < 0.001) in participants with serum albumin levels <38.0 g/L. However, when serum albumin levels were greater than or equal to 38.0 g/L, the severe impairment of ADLs no longer decreased with rising serum albumin levels.

**Conclusion:**

In summary, an L-shaped connection with an approximate inflection point of 38.0 g/L was found between blood albumin levels and significant ADL impairment in stroke patients. The results of this study suggest that increasing serum albumin levels can significantly help improve the severity of ADL impairment in stroke patients, particularly those with serum albumin levels below 38.0 g/L.

## Introduction

Stroke is the second most common cause of death globally, accounting for over 5.5 million deaths annually (1, 2). While significant advancements have been made in the treatment of stroke, the majority of patients continue to experience disability, significantly impacting their functional independence and quality of life (3). The China Stroke Surveillance Report 2021 estimates that 17.8 million adults in China had a stroke in 2020, with 2.2 million of them having disability as a result of the stroke (4). Stroke results in impairment-related functional limits that might make it difficult to do ADLs without guidance, support, or physical aid (5, 6). Thus, stroke has been the primary global cause of acquired disability in adults, and its prevalence is expected to rise (7).

Serum albumin level is a key biochemical marker of nutritional status (8–11). Lower serum albumin levels in patients indicate inadequate nutritional status and impaired physiological performance. Research suggests a connection between serum albumin and ADL (12). Specifically, Low serum albumin levels increase the risk of ADL limitation. Meanwhile, Serum albumin levels and the risk of stroke were negatively correlated (13). Zhou et al. found that hypoproteinemia was associated with neurological recovery status and mortality outcomes in patients with acute ischemic stroke or transient ischemic attack (14), but there were no outcome-related studies on ADL limitations. Improvements in nutritional status were independently associated with enhanced ADL performance during inpatient rehabilitation in older patients with malnutrition (15).

However, the association between serum albumin and severe ADL impairments in stroke patients has yet to be investigated. To fill this knowledge gap, we evaluated the relationship between severe ADL limits and serum albumin levels, as well as the dose–response relationship between the two, in stroke patients.

## Materials and methods

### Study population

This study used a cross-sectional design to gather stroke patients who were admitted to Shanghai East Hospital between January 2020 to August 2022. It comprised patients (18 years of age or older) whose diagnosis of stroke was verified by cranial CT or MRI within 24 h of admission.

**Figure 1:**
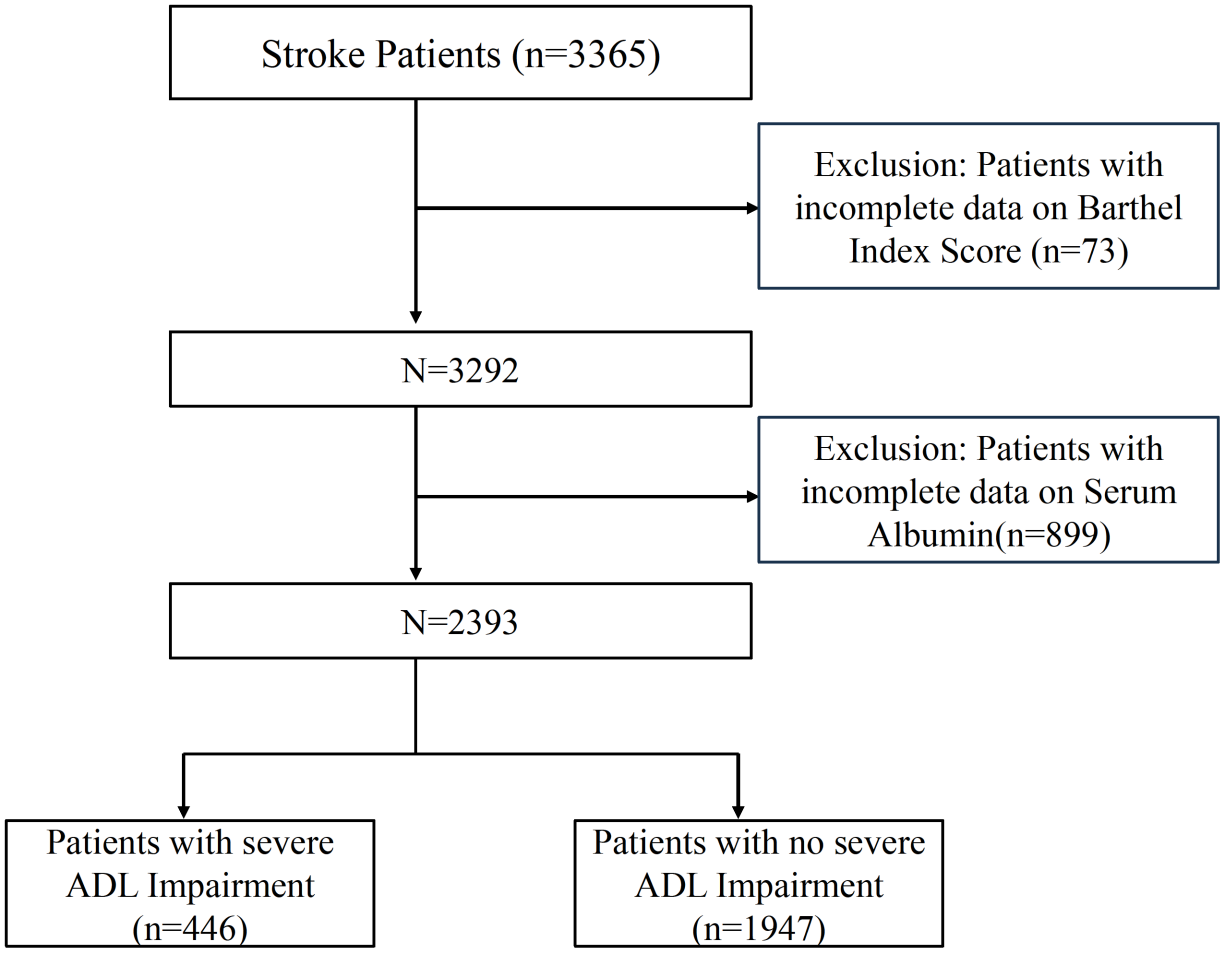
The study’s flow diagram.

Patients without a reported Barthel Index score at admission or those whose serum albumin levels were not tracked were excluded. Figure 1 illustrates the patient selection process. The cross-sectional study was reported concerning the Strengthening the Reporting of Observational Studies in Epidemiology (STROBE) guidelines and approved by the Shanghai East Hospital Ethics Committee (No. 2024055). As a retrospective cross-sectional study with anonymous data collection, informed consent was not required. All procedures and methods followed the World Medical Association’s Declaration of Helsinki on Human Experimentation ethics.

### General data collection

Participants´ demographic and lifestyle data was gathered. Individual anthropometric information was entered into the hospital’s electronic medical record system, including height, weight, sex, and age. In summary, these anthropometric measurements were taken at the time of the patient’s hospital admission during their physical examinations. Weight and height assessments followed the World Health Organization’s guidelines. Weight (kg) divided by height squared (m2) yielded the body mass index (BMI). There were two categories for smoking status: “current or ever smoking” and “current or ever drinking” for drinking status. A history of documented hypertension or blood pressure of at least two instances of ≥140 mmHg (systolic) or ≥ 90 mmHg (diastolic) following the acute phase of a stroke was used to diagnose hypertension. Information on diabetes, coronary heart disease, atrial fibrillation, cancer, and previous stroke history was extracted from the electronic medical records.

The National Institute of Health Stroke Scale (NIHSS) was used to evaluate the severity of the stroke at the time of admission (16, 17). The TOAST criteria classified ischemic strokes into five subtypes: large-artery atherosclerosis, small-vessel occlusion, cardioembolism, stroke with another identified etiology, and stroke with unknown etiology (18). Additionally, Serum albumin level was measured using a Roche analyser within 24 h of the onset of stroke. Serum albumin levels are measured using the bromocresol green (BCG) method, based on the principle that albumin specifically binds to the dye bromocresol green under acidic conditions, resulting in a color change that is quantified spectrophotometrically. The method is widely used and economical; when obtaining serum samples from patients, ensure that they are free from hemolytic, lipemic, or jaundiced interference to ensure the accuracy of the measurement.

Activities of daily living (ADL) comprise the basic actions that involve caring for one’s self and body, including personal care, mobility, and eating. The Barthel Index (BI) is a widely used scale for assessing a patient’s ability to perform basic activities of daily living (ADLs). It was first developed in 1965 by Dorothea Barthel and Florence Mahoney in the United States (19). The index evaluates ten functional areas, including feeding, bathing, dressing, toileting, mobility, walking, stair climbing, and bowel and bladder control (20, 21). The total score ranges from 0 to 100, with higher scores indicating greater independence in daily activities. The Barthel Index is extensively used to assess functional status in various conditions, particularly in the study of Stroke (22), Spinal Cord Injury (23), Dementia (24), and Parkinson’s Disease (25). Several previous studies in China have also used BI to assess ADL capacity in stroke patients (26, 27).

Previous studies have further categorized subjects into two groups based on activities of daily living (ADL): those with a high ADL score of 40 or more and those with a low ADL score of 40 or less (28). When any patient’s Barthel score is less than 40, it means that neither their mobility abilities nor ability to feed themselves, groom themselves, or control their sphincters are autonomous (29). The patient cannot perform daily tasks independently and relies on assistance from others. This level of impairment is defined as severe impairment of activities of daily living.

### Statistical analysis

Every patient was the subject of a descriptive analysis. Numbers (percentages) were used to express categorical data, and depending on the distribution, continuous data were shown as the median (interquartile range) or the mean ± standard deviation. To evaluate differences among groups, one-way analysis of variance (for normally distributed data), Kruskal–Wallis tests (for non-normally distributed data), and chi-square tests (for categorical variables) were performed. The odds ratios (OR) and 95% confidence intervals (CIs) for the association between serum albumin and severe impairment of ADL in stroke patients were calculated using logistic regression models. Model 1 was calibrated for BMI, age, sex, drinking, and smoking status. Age, sex, BMI, drinking and smoking status, hypertension, diabetes, cancer, atrial fibrillation, coronary heart disease, and history of stroke were all taken into account while adjusting Model 2. All of the variables from Model 2 were included in Model 3, along with TOAST classification and NIHSS.

Additionally, after controlling for the factors in Model 3, we used restricted cubic spline (RCS) regression to analyze curvilinearity and investigate the dose–response association between serum albumin and severe ADL impairment. To evaluate the association threshold between serum albumin and severe ADL impairment after stroke, we employed a smoothed binary logistic regression model. Likelihood ratio tests and bootstrap regression were used to identify significant inflection points in this relationship.

Furthermore, we investigated several variables that could alter the association between serum albumin and severe ADL impairment following a stroke. The variables that were analyzed were sex; age (less than 60 years old versus more than 60 years old); BMI (less than 25, 25–29.9, versus more than 30 kg/m^2^); smoking and drinking status (yes or no); hypertension (yes or no); diabetes (yes or no); and TOAST classification (large-artery atherosclerosis, small-vessel occlusion, cardioembolism, stroke with another identified etiology, and stroke with unknown etiology).

Using multivariate logistic regression, we assessed subgroup heterogeneity, and the likelihood ratio test examine interactions between subgroups and serum albumin.

To examine the robustness of the results, we did a sensitivity analysis by interpolating the NIHSS, which had a high number of missing data, using median values. No, *a priori* statistical power analyses were carried out because the sample size was established exclusively using the data that were available data. R version 4.3.1^1^ and Free Statistics version 1.8 were used for all analyses. A descriptive study was carried out on each individual. A two-tailed *p*-value of less than 0.05 was specified as statistically significant.

## Results

### Study population

The study initially included 3,365 stroke patients. Among these, 73 patients were eliminated due to missing data on ADL, whereas 899 patients were omitted due to missing albumin levels. As a result, this cross-sectional study comprised 2,393 patients from Shanghai East Hospital from 2020 to 2022. Figure 1 provides a detailed illustration of the selection process, depicting both inclusion and exclusion criteria.

### Baseline characteristics

Table 1 summarizes all subjects´ baseline characteristics, classified by serum albumin quartiles.

**Table 1:** The baseline characteristics by categories of serum albumin.

Variables	Total	Q1 (<37.4)	Q2 (37.4–40.21)	Q3 (40.21–42.80)	Q4 (≥42.8)	*p*
No.	2,393	597	599	590	607	
Sex, n (%)						< 0.001
Male	1,538 (64.3)	347 (58.1)	365 (60.9)	388 (65.8)	438 (72.2)	
Female	855 (35.7)	250 (41.9)	234 (39.1)	202 (34.2)	169 (27.8)	
Age, Mean ± SD	69.6 ± 11.6	74.6 ± 11.0	71.5 ± 10.1	68.0 ± 10.8	64.3 ± 11.8	< 0.001
BMI, Mean ± SD	24.5 ± 3.4	23.7 ± 3.6	24.4 ± 3.5	24.8 ± 3.3	25.0 ± 3.2	< 0.001
Barthel Index Score, Mean ± SD	62.1 ± 26.9	49.2 ± 27.8	61.2 ± 26.0	68.2 ± 23.6	69.9 ± 25.1	< 0.001
NIHSS, Mean ± SD	3.9 ± 4.7	5.5 ± 5.7	3.7 ± 4.1	3.2 ± 3.5	3.4 ± 5.0	< 0.001
Hypertension, *n* (%)						0.028
No	472 (20.2)	139 (24.3)	119 (20.2)	109 (18.8)	105 (17.6)	
Yes	1867 (79.8)	434 (75.7)	470 (79.8)	471 (81.2)	492 (82.4)	
Diabetes, *n* (%)						0.27
No	1,257 (53.7)	328 (57.2)	309 (52.5)	302 (52.1)	318 (53.3)	
Yes	1,082 (46.3)	245 (42.8)	280 (47.5)	278 (47.9)	279 (46.7)	
Coronary heart disease, *n* (%)						< 0.001
No	1952 (83.5)	448 (78.2)	486 (82.5)	501 (86.4)	517 (86.6)	
Yes	387 (16.5)	125 (21.8)	103 (17.5)	79 (13.6)	80 (13.4)	
Atrial fibrillation, *n* (%)						< 0.001
No	2053 (87.8)	444 (77.5)	509 (86.4)	539 (92.9)	561 (94)	
Yes	286 (12.2)	129 (22.5)	80 (13.6)	41 (7.1)	36 (6)	
Cancer, *n* (%)						0.374
No	2048 (87.6)	498 (86.9)	507 (86.1)	518 (89.3)	525 (87.9)	
Yes	291 (12.4)	75 (13.1)	82 (13.9)	62 (10.7)	72 (12.1)	
History of stroke, *n* (%)						0.951
No	1934 (82.7)	471 (82.2)	490 (83.2)	477 (82.2)	496 (83.1)	
Yes	405 (17.3)	102 (17.8)	99 (16.8)	103 (17.8)	101 (16.9)	
Smoking status, *n* (%)						0.002
No	1,289 (58.7)	362 (64.9)	320 (59.1)	307 (57.1)	300 (53.8)	
Yes	906 (41.3)	196 (35.1)	221 (40.9)	231 (42.9)	258 (46.2)	
Drinking status, *n* (%)						0.562
No	1887 (82.7)	469 (83.3)	471 (82.8)	478 (83.9)	469 (80.9)	
Yes	395 (17.3)	94 (16.7)	98 (17.2)	92 (16.1)	111 (19.1)	
TOAST classification, *n* (%)						< 0.001
Large-artery atherosclerosis	865 (42.4)	209 (43.7)	211 (40.1)	212 (41.7)	233 (44)	
Small-vessel occlusion	802 (39.3)	133 (27.8)	212 (40.3)	231 (45.4)	226 (42.7)	
Cardioembolism	204 (10.0)	76 (15.9)	66 (12.5)	33 (6.5)	29 (5.5)	
Stroke of another determined etiology	55 (2.7)	20 (4.2)	12 (2.3)	9 (1.8)	14 (2.6)	
Stroke of unknown etiology	116 (5.7)	40 (8.4)	25 (4.8)	24 (4.7)	27 (5.1)	

The average age of the patients was 69.6 ± 11.6 years, with 1,538 (64.3%) being male. Patients with higher serum albumin levels were younger, male, had a slightly higher BMI, higher BI scores, lower NIHSS scores, had a history of hypertension, no history of coronary heart disease and atrial fibrillation, were non-smokers, and had atherosclerosis according to the TOAST classification. The four groups had significant differences in sex, age, BMI, Barthel Index Score, NIHSS, Hypertension, Coronary heart disease, Atrial Fibrillation, Smoking Status, and TOAST (all *p* < 0.05).

### Relationship between serum albumin and severe impairment of ADL among stroke patients

After adjusting for confounding factors, the multivariate analysis revealed a 7% decrease in severe impairment of ADL after stroke for every unit (g/L) increase in serum albumin levels. When blood albumin levels were analyzed using quartiles, a negative relationship was found between serum albumin levels and severe impairment of ADL after controlling for relevant variables. In comparison to patients with lower levels of serum albumin (<37.4 g/L), the adjusted odds ratio (OR) for severe impairment of ADL after stroke for those in the second quartile (Q2: 37.4–40.21 g/L), third quartile (Q3: 40.21–42.80 g/L), and fourth quartile (Q4: >42.8 g/L) were 0.68 (95% confidence interval [CI]: 0.40–1.15, *p* = 0.148), 0.55 (95% CI: 0.32–0.97, *p* = 0.04), and 0.65 (95% CI: 0.37–1.15, *p* = 0.139), respectively (Table 2).

**Table 2:** Association between serum albumin and severe impairment of ADL among stroke patients.

Variable	n. total	n. event_%	Crude model		Model 1		Model 2		Model 3	
			OR (95%CIs)	*p*-value	OR (95%CIs)	*p*-value	OR (95%CIs)	*p*-value	OR (95%CIs)	*p*-value
Albumin (g/L)	2,393	446 (18.6)	0.85 (0.83 ~ 0.87)	<0.001	0.9 (0.87 ~ 0.93)	<0.001	0.9 (0.87 ~ 0.94)	<0.001	0.93 (0.88 ~ 0.98)	0.004
Albumin Group (g/L)										
Q1 (<37.4)	597	212 (35.5)	1 (Ref)		1 (Ref)		1 (Ref)		1 (Ref)	
Q2 (37.4–40.21)	599	108 (18)	0.4 (0.31 ~ 0.52)	<0.001	0.49 (0.34 ~ 0.71)	<0.001	0.5 (0.34 ~ 0.73)	<0.001	0.68 (0.4 ~ 1.15)	0.148
Q3 (40.21–42.80)	590	58 (9.8)	0.2 (0.14 ~ 0.27)	<0.001	0.43 (0.29 ~ 0.64)	<0.001	0.45 (0.3 ~ 0.68)	<0.001	0.55 (0.32 ~ 0.97)	0.04
Q4 (≥42.8)	607	68 (11.2)	0.23 (0.17 ~ 0.31)	<0.001	0.44 (0.29 ~ 0.66)	<0.001	0.48 (0.31 ~ 0.73)	0.001	0.65 (0.37 ~ 1.15)	0.139
Trend test				<0.001		<0.001		<0.001		0.116

Q, quartiles; OR, odds ratio; CI, confidence interval; Ref: reference. Model 1 was adjusted for Sex, Age, BMI, Smoking Status, and Drinking Status. Model 2 was adjusted for Sex, Age, BMI, Smoking Status, Drinking Status, Hypertension, Diabetes, Coronary heart disease, Atrial fibrillation, Cancer, and History of stroke. Model 3 was adjusted for Sex, Age, BMI, Smoking Status, Drinking Status, Hypertension, Diabetes, Coronary heart disease, Atrial fibrillation, Cancer, and History of stroke, NIHSS, TOAST classification.

**Figure 2:**
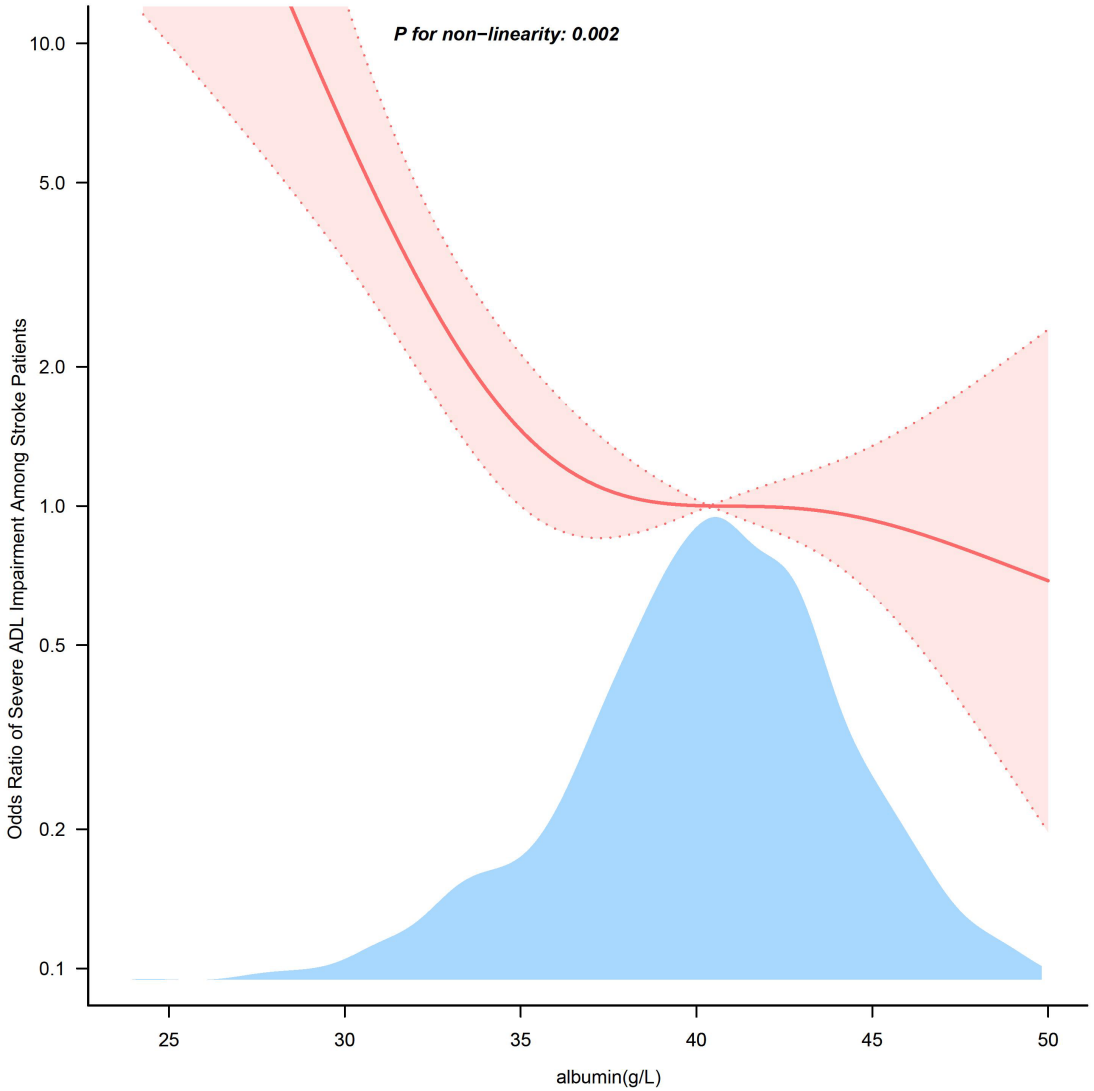
Association between serum albumin levels and the odds ratio of severe ADL impairment among stroke patients. Solid and dashed lines represent the predicted value and 95% confidence intervals. They were adjusted for Sex, Age, BMI, Smoking Status, and Drinking Status, Hypertension, Diabetes, Coronary heart disease, Atrial fibrillation, Cancer, and History of stroke, NIHSS, TOAST. Only 99% of the data is displayed.

In Figure 2, the link between serum albumin and severe impairment of ADLs after stroke displayed an L-shaped curve (nonlinear, *p* = 0.002). The link between serum albumin levels and severe impairment of ADLs among stroke patients shows an inflection point around 38.0 g/L. In the threshold analysis, the odds ratio (OR) for severe impairment of ADLs among stroke patients with serum albumin levels less than 38.0 g/L was 0.680 (95% CI: 0.568–0.814, *p* < 0.001). When serum albumin levels were greater than or equal to 38.0 g/L, the severe impairment of ADLs no longer decreased with rising serum albumin levels, indicating that the threshold had been reached (Table 3).

**Table 3:** Threshold effect analysis of the relationship of serum albumin with severe impairment of ADL among stroke patients.

Serum albumin (g/L)	Adjusted Model	
	OR (95%CI)	*p*-value
<38.0	0.680 (0.568 ~ 0.814)	<0.001
≥38.0	0.961 (0.875 ~ 1.057)	0.4125
Likelihood Ratio test		0.001

OR, odds ratio; CI, confidence interval. Adjusted for Sex, Age, BMI, Smoking Status, and Drinking Status, Hypertension, Diabetes, Coronary heart disease, Atrial fibrillation, Cancer, and History of stroke, NIHSS, TOAST classification. Only 99% of the data is displayed.

### Subgroup analyses

**Figure 3:**
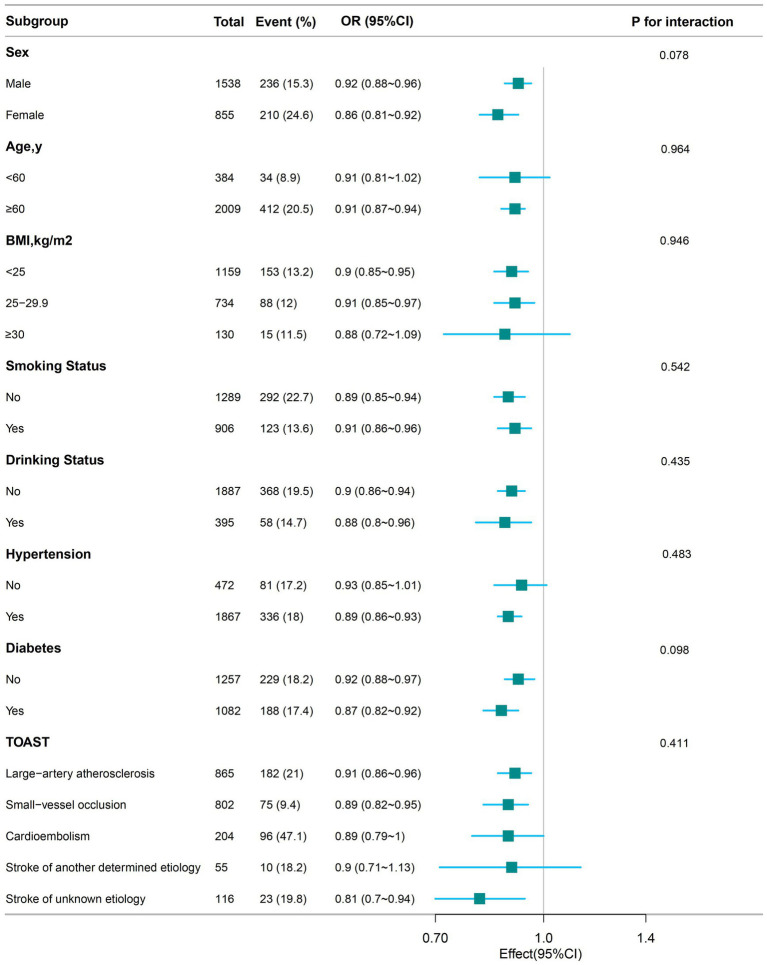
The relationship between serum albumin levels and the odds ratio of severe ADL impairment among stroke patients according to basic features. Except for the stratification component itself, each stratification factor was adjusted for all other variables (Sex, Age, BMI, Smoking Status, Drinking Status, Hypertension, Diabetes, Coronary heart disease, Atrial fibrillation, Cancer, History of stroke, NIHSS, TOAST).

Figure 3 illustrates the relationship between serum albumin levels and severe limitations in ADL, as analyzed across various subgroups. Serum albumin levels were associated with severely impaired ability to perform activities of daily living in male (OR, 0.92; 95% CI, 0.88–0.96), female (OR, 0.86; 95% CI, 0.81–0.92), more than 60 years (OR, 0.91; 95% CI, 0.87–0.94), with BMI less than 25 kg/cm^2^ (OR, 0.9; 95% CI, 0.85–0.95), BMI 25–29.9 kg/cm^2^ (OR, 0.91; 95% CI, 0.85–0.97), smoking (OR, 0.91; 95% CI, 0.86–0.96), non-smoking (OR, 0.89; 95% CI, 0.85–0.94), alcohol (OR, 0.88; 95% CI, 0.8–0.96), no alcohol (OR, 0.9; 95% CI, 0.86–0.94), hypertension (OR, 0.89; 95% CI, 0.86–0.93), diabetes (OR, 0.91; 95% CI, 0.85–0.97), no diabetes (OR, 0.92; 95% CI, 0.88–0.97), and those with TOAST classification large-artery atherosclerosis (OR, 0.91; 95% CI, 0.86–0.96), small-artery occlusion (OR, 0.89; 95% CI, 0.82–0.95), stroke of unknown etiology (OR, 0.81; 95% CI, 0.7–0.94). There was no significantly association among aged less than 60 years, BMI more than 30 kg/cm^2^, or those without hypertension or in the Cardioembolism and Stroke of another determined etiology subgroups. After subgroup analysis according to sex, age, BMI, smoking status, alcohol status, hypertension, and diabetes, TOAST classification, no significant interactions were found in any subgroup (all *p*-values for interaction >0.05).

### Sensitivity analysis

Due to the high rate of missing NIHSS and TOAST data, we found a more robust association between serum albumin and severely impaired ADL of stroke after the multiple interpolation of NIHSS and TOAST. After adjusting for confounding factors, multivariate analysis showed that for every 1 g/L increase in serum albumin levels, there was a 7% reduction in severe ADL impairment post-stroke. Compared with those with lower serum albumin levels (<37.4 g/L), the adjusted ORs for serum albumin and ADL in Q2 (37.4–40.21 g/L), Q3 (40.21–42.80 g/L), and Q4 (>42.8 g/L) were 0.57 (95% CI: 0.38–0.86, *p* = 0.007), 0.50 (95% CI: 0.32–0.77, *p* = 0.002), and 0.52 (95% CI: 0.33–0.81, *p* = 0.004), respectively (Supplementary Table S1).

## Discussion

Serum albumin level significantly indicates an individual’s ability to perform ADL (30). Studies have shown that serum albumin levels are associated with physical functioning disability among older adults (31). Additionally, higher serum albumin levels in ischemic stroke patients are associated with a lower likelihood of poor prognosis (32, 33). Due to its relatively long half-life, serum albumin reflects a patient’s nutritional status before stroke onset. Albumin levels measured within 24 h of admission are less likely to be affected by the acute stress of stroke (34). Consequently, this study collected albumin levels within 24 h of admission. The results revealed an L-shaped relationship between serum albumin levels and severely impaired ADL in stroke patients, with an inflection point at 38.0 g/L. Subgroup analyses revealed that serum albumin levels were inversely associated with severe ADL limitations following a stroke in different subgroups, consistent with the primary outcome. In this study, serum albumin was not significantly associated with severe ADL impairment in patients younger than 60 years, those with BMI greater than 30, individuals without hypertension, and with cardioembolism or stroke of another determined etiology. Given the limited sample size in these subgroups, caution is warranted in interpreting these findings, and further well-designed prospective studies are needed to confirm these results. Subgroup analysis stratified by sex, age, BMI, smoking status, alcohol status, hypertension, diabetes, and TOAST classification showed no significant interactions in any subgroup. Sensitivity analyses also reinforced the robustness of the observed relationship between serum albumin levels and severe ADL impairment after stroke.

Recent studies have confirmed a strong association between blood albumin levels and ADL in the general old population (9, 35–44). A cross-sectional study of centenarians demonstrated that albumin levels were negatively associated with the likelihood of ADL disability. Higher albumin levels correlated with a lower likelihood of ADL disability, suggesting a protective effect of albumin against ADL decline in centenarians (45). However, the mechanisms connecting serum albumin to daily mobility after stroke remain unclear.

There are three possible mechanisms by which albumin enhances daily mobility after stroke. First, during the early reperfusion phase in acute ischemic stroke, albumin antagonizes stagnation, thrombosis, and leukocyte adhesion within the postcapillary microcirculation (8). Adequate serum albumin amounts increase microcirculatory flow, plasma viscosity, and oxygen transport capacity (46). Second, albumin may protect nerves by reducing cerebral edema or through its antioxidant or anti-apoptotic properties (47). Third, serum albumin levels have a favorable effect on the immune system (46), as protein energy deficiency after acute stroke impairs cellular immune function and further worsens prognosis, thereby increasing the risk of poor prognosis.

The strength of our study lies in its novel exploration of the association between serum albumin levels and severe ADL impairment following stroke, including the first discussion of their dose–response relationship. However, several limitations must be considered. Firstly, our data was exclusively obtained from 2020 to 2022. To ensure greater consistency, a larger dataset would be essential. Secondly, despite utilizing regression modeling, stratified analyses, and sensitivity analyses, residual confounding effects from unmeasured or unknown factors cannot be entirely excluded. Thirdly, our findings are based on a survey conducted with adults, necessitating further research to confirm their applicability to other populations. Finally, the cross-sectional nature of our study limits our ability to establish a causal relationship between serum albumin and severe ADL impairment after stroke, which requires further investigation through longitudinal studies. Additionally, future research could explore other biochemical markers that may influence ADL impairment post-stroke.

## Conclusion

To summarize, our study found an L-shaped correlation between serum albumin levels and severe ADL impairment in stroke patients, with a critical threshold at around 38.0 g/L. These findings indicate that elevating serum albumin levels can notably reduce the severity of ADL impairment, especially in patients whose serum albumin levels are below the 38.0 g/L mark.

## Data Availability

The original contributions presented in the study are included in the article/Supplementary material, further inquiries can be directed to the corresponding author.

## Footnotes

^1^http://www.R-project.org, The R Foundation.
