# Supplementary material for: Association between serum albumin and severe impairment of activities of daily living in patients with stroke: a cross-sectional study
Source: Front Neurol. 2025 Jan 6;15:1501294. doi: 10.3389/fneur.2024.1501294 (PMC11743378; doi:10.3389/fneur.2024.1501294)
Supplement: Supplementary file 1 [file Table_1.docx]

**Supplementary Table S1. Association between serum albumin and severe impairment of ADL among stroke patients after the multiple interpolation of NIHSS and TOAST**

| Variable | n. total | n. event（%） | Crude model | | Model 1 | | Model 2 | | Model 3 | |
| --- | --- | --- | --- | --- | --- | --- | --- | --- | --- | --- |
|  |  |  | OR（95%CIs） | *P* value | OR（95%CIs） | *P* value | OR（95%CIs） | P-value | OR（95%CIs） | P-value |
| Albumin（g/L） | 2393 | 446 (18.6) | 0.85 (0.83~0.88) | <0.001 | 0.91 (0.88~0.94) | <0.001 | 0.92 (0.89~0.95) | <0.001 | 0.93 (0.89~0.96) | <0.001 |
| Albumin Group（g/L） |  |  |  |  |  |  |  |  |  |  |
| Q1（＜37.4） | 597 | 212 (35.5) | 1(Ref) |  | 1(Ref) |  | 1(Ref) |  | 1(Ref) |  |
| Q2（37.4-40.21） | 599 | 108 (18) | 0.40 (0.31~0.52) | <0.001 | 0.49 (0.34~0.71) | <0.001 | 0.50 (0.34~0.73) | <0.001 | 0.57 (0.38~0.86) | 0.007 |
| Q3 (40.21-42.80） | 589 | 57 (9.7) | 0.19 (0.14~0.27) | <0.001 | 0.43 (0.29~0.63) | <0.001 | 0.44 (0.29~0.66) | <0.001 | 0.50 (0.32~0.77) | 0.002 |
| Q4（≥42.8） | 608 | 69 (11.3) | 0.23 (0.17~0.31) | <0.001 | 0.45 (0.30~0.68) | <0.001 | 0.49 (0.32~0.75) | 0.001 | 0.52 (0.33~0.81) | 0.004 |
| Trend test |  |  |  | <0.001 |  | <0.001 |  | <0.001 |  | 0.002 |

Q, quartiles; OR, odds ratio; CI, confidence interval; Ref: reference.

Model 1 was adjusted for Sex, Age, BMI, Smoking Status, and Drinking Status.

Model 2 was adjusted for Sex, Age, BMI, Smoking Status, and Drinking Status, Hypertension, Diabetes, Coronary heart disease, Atrial fibrillation, Cancer, and History of stroke.

Model 3 was adjusted for Sex, Age, BMI, Smoking Status, and Drinking Status, Hypertension, Diabetes, Coronary heart disease, Atrial fibrillation, Cancer, and History of stroke, NIHSS, TOAST.
